# Editorial: Non-Coding RNAs in Breast Cancer

**DOI:** 10.3389/fonc.2021.789798

**Published:** 2021-11-11

**Authors:** Wenwen Zhang, Naoyuki Kataoka, Xiaoxiang Guan

**Affiliations:** ^1^ Department of Oncology, Nanjing First Hospital, Nanjing Medical University, Nanjing, China; ^2^ Laboratory of Cellular Biochemistry, Department of Animal Resource Sciences, Graduate School of Agriculture and Life Sciences, The University of Tokyo, Tokyo, Japan; ^3^ Department of Oncology, The First Affiliated Hospital of Nanjing Medical University, Nanjing, China; ^4^ Jiangsu Key Lab of Cancer Biomarkers, Prevention and Treatment, Collaborative Innovation Center for Personalized Cancer Medicine, Nanjing Medical University, Nanjing, China

**Keywords:** non-coding RNAs, breast cancer, ceRNA, therapeutic targets, biomarkers

Breast cancer (BC) is a highly heterogeneous disease and the most common malignancy in women worldwide. Despite early diagnosis and comprehensive treatment, including endocrine therapy, molecular targeted therapy and emerging immunotherapy, breast cancer mortality remains relatively high (1). Thus, identification of novel biomarkers for therapeutic targets and efficacy prediction in breast cancer is urgently required. Apart from about 2% protein-coding genes, the vast majority of the human genome is made up of non-coding RNA (ncRNA), including microRNAs (miRNAs), long non-coding RNAs (lncRNAs), circular RNA (circRNA), small nucleolar RNA (snoRNA), and PIWI-interacting RNAs (piRNA). To date, a large number of ncRNAs have been identified and found to be dysregulated in various types of cancers including breast cancer. The biological functions of ncRNAs have been extensively studied, and several ncRNAs have been reported to play important roles in various biological processes of breast cancer, including cell proliferation, apoptosis, migration, invasion, angiogenesis, and drug resistance. In addition, the potential of ncRNAs as diagnostic, prognostic biomarkers, and therapeutic targets has been extensively explored in breast cancer (2–4). This Research Topic collected 10 scientific studies (six original research articles, and four reviews), which focused on the new findings or reviewed recent advances of ncRNAs in breast cancer.

miRNAs and lncRNAs are the two most widely studied ncRNAs in breast cancer. miRNAs are small noncoding RNAs of approximately 19 to 25 nucleotides, that can modulate gene expression by targeting selective mRNAs sequences, inducing translational repression or mRNA degradation. Tommasi et al. summarized the mechanisms by which miRNAs interact with BRCA genes, and the role of miRNAs in influencing the risk and diagnosis of BRCA-related breast cancer. They also discussed the biological and clinical significance of the link between nutritional and lifestyle interventions, miRNA expression and germline BRCA mutations. Pedroza et al. performed a whole human miRNome profiling to identify altered miRNAs and miRNA-mRNA network hubs, after AG-205 treatment and PGRMC1 silencing in the TNBC cell line MDA-MB-468. Enrichment analysis showed that the target genes of PGRMC1-altered miRNAs were uniquely involved in signaling pathways, including pathways in cancer, cell cycle and p53 signaling pathway.

LncRNAs are untranslated transcripts with a length of 200 nucleotides or more. Recently, lncRNAs have been reported to have multiple regulatory functions, including acting as regulators of transcription and chromatin remodeling, splicing factors, regulators of mRNA stability, protein decoys and miRNA sponges (5, 6). Among them, lncRNAs are widely studied to act as molecular sponges of miRNAs that compete for miRNA-targeted mRNAs, thereby forming a complex post-transcriptional regulatory network, called the competitive endogenous RNAs (ceRNA) network. In this research topic, several reports have shown that sponge lncRNAs play a critical role in regulating the cancer initiation, progression and drug resistance of breast cancer. Li et al. identified LINC01977 as a key oncogenic driver, that promotes breast cancer progression and chemoresistance to doxorubicin by targeting the miR-212-3p/GOLM1 axis. Cisneros-Villanueva et al. observed that the LINC00460 expression is significantly enriched in the basal-like 2 (BL2) triple negative breast cancer (TNBC) subtype. LINC00460 potentially binds to miR-103-a-1, and acts as a potential regulator of WNT7A expression, resulting in activating the WNT differentiation pathway. Moreover, they also found that the LINC00460:WNT7A ratio could serve as a composite marker to predict favorable overall survival (OS) and distant metastasis-free survival (DMFS) in TNBC, and that their combined expression could predict anthracycline therapy response in ER-positive breast cancer patients.

Other two studies constructed ceRNA regulatory network using differentially expressed lncRNAs, miRNAs, and mRNAs from Gene Expression Omnibus (GEO) or The Cancer Genome Atlas (TCGA) databases. Qin et al. established a new ceRNA regulatory network in TNBC, based on six lncRNAs, 295 miRNAs, and 573 mRNAs. They developed a predictive model for recurrence and pathological stage of TNBC patients, on the basis of a prognostic scoring model of eight differentially expressed genes. Besides, they also constructed a network of small-molecule drugs targeting these eight differentially expressed genes to predict potential therapeutic agents. Another study established a novel strategy to construct a cell-specific ceRNA network to explore the function of hub lncRNAs in the regulation of estrogen in breast cancer. Chen et al. built a cell-specific RNA-RNA co-expression network, based on single-cell expression profiles of predefined reference cells. Next, they constructed a cell-specific ceRNA network to specify breast cancer cell subtypes, by integrating the cell-specific RNA-RNA co-expression network with the existing ceRNA network. They found that NEAT1 is a hub lncRNA of the early estrogen response subtype, and lncRNA DLEU2 is potentially involved in GPCR signaling.

Several authors focused on other ncRNAs in this Research Topic. Circular RNAs (circRNAs), structurally stable non-coding RNAs with a covalently closed circular structure, have been identified and shown to play an important role in the development and progression of breast cancer. Xu et al. summarized recent advances in the regulatory network of circRNA biogenesis, degradation and distribution, as well as the functions, mechanisms and clinical significances of circRNA in breast cancer. PIWI-interacting RNAs (piRNAs) were reported to bind with PIWI family proteins to form PIWI–piRNA complexes to regulate gene expression at the epigenetic and post-transcriptional levels. Qian et al. reviewed the advances, challenges and perspectives of oncogenic or tumor suppressor piRNAs and their regulatory mechanisms in breast cancer.

In addition, two articles provide new research approaches to explore the role of ncRNAs in breast cancer. Mathias et al. proposed a novel bioinformatic approach to incorporate lncRNAs complexity into breast cancer molecular and immune subtypes, by using signal-to-noise ratio metrics to build these subtype-specific signatures. They obtained five immune-related signatures from approximately ten specific lncRNAs, which act as regulators of the immune response, and are associated with different breast cancer specific molecular subtypes. Such as, MEG3, EBLN3P, XXYLT1-AS2, LINC01871, and LINC02613 were associated with immune response activation (or suppression) in Luminal A, Luminal B, HER2-enriched, basal-like and normal-like subtypes, respectively. Animal xenotransplantation means the implantation of human tumor cells into animal hosts for *in vivo* monitoring of tumor development, elucidating pathogenesis and designing new therapeutic strategies. Mouse xenotransplantation is the most commonly used animal model, but the cost and complexity of raising mice are problems that have to be considered. The zebrafish xenograft model offers the accessibility of a xenograft assay as well as economic and experimental advantages. Zampedri et al. summarized the advantages of zebrafish xenotransplants compared to other models, and the use of zebrafish xenotransplants to study the role of lncRNAs in breast cancer development, including proliferation, differentiation, migration, metastasis, angiogenesis and response to drugs.

Taken together, all these studies in the present Research Topic provide new insights into the role of ncRNAs during the development and progression of breast cancer. However, the potential of ncRNAs as therapeutic targets for breast cancer should be more extensively explored in the future. We hope that ncRNAs-based therapeutics would soon evolve into viable options for the treatment of breast cancer patients, either alone or in combination with existing therapeutic agents.

## Author Contributions

All authors contributed equally to this Editorial. All authors contributed to the article and approved the submitted version.

## Funding

This research was supported by National Natural Science Foundation of China (No. 81773102 to XG, 81802667 to WZ), Natural Science Foundation of Jiangsu Province (BK20180133 to WZ), Nanjing Outstanding Youth Fund (No. JQX20009 to WZ), Key International Cooperation of the National Natural Science Foundation of China (No. 81920108029 to XG), Key Foundation for Social Development Project of the Jiangsu Province, China (BE2021741 to XG), and Grants-in-Aid for Scientific Research (18K06012 to NK).

## Conflict of Interest

The authors declare that the research was conducted in the absence of any commercial or financial relationships that could be construed as a potential conflict of interest.

## Publisher’s Note

All claims expressed in this article are solely those of the authors and do not necessarily represent those of their affiliated organizations, or those of the publisher, the editors and the reviewers. Any product that may be evaluated in this article, or claim that may be made by its manufacturer, is not guaranteed or endorsed by the publisher.

## References

[1] Siegel RL Miller KD Fuchs HE Jemal A. Cancer Statistics, 2021. CA Cancer J Clin (2021) 71(1):7–33. doi: 10.3322/caac.21654 33433946

[2] Zhang W Guan X and Tang J. The Long non-Coding RNA Landscape in Triple-Negative Breast Cancer. Cell Prolif (2021) 54(2):e12966. doi: 10.1111/cpr.12966 33314471 PMC7848969

[3] Dsouza VL Adiga D Sriharikrishnaa S Suresh PS Chatterjee A Kabekkodu SP. Small Nucleolar RNA and Its Potential Role in Breast Cancer - A Comprehensive Review. Biochim Biophys Acta Rev Cancer (2021) 1875(1):188501. doi: 10.1016/j.bbcan.2020.188501 33400969

[4] Kandettu A Radhakrishnan R Chakrabarty S Sriharikrishnaa S Kabekkodu SP. The Emerging Role of miRNA Clusters in Breast Cancer Progression. Biochim Biophys Acta Rev Cancer (2020) 1874(2):188413. doi: 10.1016/j.bbcan.2020.188413 32827583

[5] Palazzo AF Koonin EV. Functional Long Non-Coding RNAs Evolve From Junk Transcripts. Cell (2020) 183(5):1151–61. doi: 10.1016/j.cell.2020.09.047 33068526

[6] Liu H Luo J Luan S He C and Li Z. Long non-Coding RNAs Involved in Cancer Metabolic Reprogramming. Cell Mol Life Sci (2019) 76(3):495–504. doi: 10.1007/s00018-018-2946-1 30341461 PMC11105355

